# Bayesian phylogeography of influenza A/H3N2 for the 2014-15 season in the United States using three frameworks of ancestral state reconstruction

**DOI:** 10.1371/journal.pcbi.1005389

**Published:** 2017-02-07

**Authors:** Daniel Magee, Marc A. Suchard, Matthew Scotch

**Affiliations:** 1 Department of Biomedical Informatics, Arizona State University, Tempe, Arizona, United States of America; 2 Biodesign Center for Environmental Security, Arizona State University, Tempe, Arizona, United States of America; 3 Departments of Biomathematics and Human Genetics, David Geffen School of Medicine, University of California, Los Angeles, California, United States of America; 4 Department of Biostatistics, School of Public Health, University of California, Los Angeles, California, United States of America; Duke University, UNITED STATES

## Abstract

Ancestral state reconstructions in Bayesian phylogeography of virus pandemics have been improved by utilizing a Bayesian stochastic search variable selection (BSSVS) framework. Recently, this framework has been extended to model the transition rate matrix between discrete states as a generalized linear model (GLM) of genetic, geographic, demographic, and environmental predictors of interest to the virus and incorporating BSSVS to estimate the posterior inclusion probabilities of each predictor. Although the latter appears to enhance the biological validity of ancestral state reconstruction, there has yet to be a comparison of phylogenies created by the two methods. In this paper, we compare these two methods, while also using a primitive method without BSSVS, and highlight the differences in phylogenies created by each. We test six coalescent priors and six random sequence samples of H3N2 influenza during the 2014–15 flu season in the U.S. We show that the GLMs yield significantly greater root state posterior probabilities than the two alternative methods under five of the six priors, and significantly greater Kullback-Leibler divergence values than the two alternative methods under all priors. Furthermore, the GLMs strongly implicate temperature and precipitation as driving forces of this flu season and nearly unanimously identified a single root state, which exhibits the most tropical climate during a typical flu season in the U.S. The GLM, however, appears to be highly susceptible to sampling bias compared with the other methods, which casts doubt on whether its reconstructions should be favored over those created by alternate methods. We report that a BSSVS approach with a Poisson prior demonstrates less bias toward sample size under certain conditions than the GLMs or primitive models, and believe that the connection between reconstruction method and sampling bias warrants further investigation.

## Author summary

For the better part of the last decade, epidemiological researchers have employed a Bayesian framework to reconstruct phylogenetic trees and determine the spatiotemporal relationships between clades of viruses. Recently, an extension of this framework has enabled direct assessment of how various demographic, geographic, genetic, and environmental variables play a role in these relationships, but there has yet to be a comparison between the former and the latter. Here, we aim to assess the differences between the two reconstruction techniques, as well as an additional primitive method, using the 2014–15 influenza season in the U.S. as a case study under a variety of population growth scenarios. We highlight how the new method demonstrates significant increases in commonly-reported trends in phylogenies and that the method identifies climate predictors that appear to be consistent with known trends in seasonal trends in influenza. However, we found that this method appears to be the most heavily influenced by the locations at which the viruses were obtained. Our work offers valuable insight for researchers wishing to study the evolutionary history of viruses and also may prove useful in determining the correct method to choose for a given application of virus phylogeography.

## Introduction

Bayesian phylogeography has emerged as a powerful approach to analyzing virus spread. It utilizes sequence data to perform ancestral reconstruction and estimate the most likely lineages of the viruses in rooted, time-measured phylogenies [1] using nucleotide substitution models, molecular clocks, and coalescent priors under a probabilistic Bayesian framework known as Bayesian stochastic search variable selection (BSSVS) [1–3]. This framework has improved ancestral state reconstruction and has recently been used to analyze human and animal influenza viruses both globally [4–5] and nationally [6–7]. By identifying the relationship between geospatial origins and genetic lineages, much can be learned about the complex process in which these viruses spread. Phylodynamic analyses that aim to combine immunological, epidemiological, and evolutionary biology techniques [8] also enhance our understanding of virus transmission dynamics and their relationship to a phylogeny. These studies have unveiled novel properties of several influenza viruses, including pdm09 [9], H3N2 [10] and highly pathogenic avian influenza H5N1 [11]. Building upon the benefits of a BSSVS framework, recent work by Lemey *et al*. [12] utilized a phylogeographic generalized linear model (GLM) approach to identify environmental, genetic, demographic, and geographic predictors that contributed to the global spread of H3N2 influenza viruses. In the GLM, the BSSVS on the discrete location variable is instead used to estimate the posterior inclusion probability of potential predictors in a log-linear combination to model the transition rate matrix. Similarly, studies have followed this approach to uncover the predictors associated with the spread of H5N1 in Egypt [13] and for HIV in Brazil [14]. Such studies have demonstrated the utility of combining genetic and geospatial inferences from phylogeography with surveillance data in epidemiological studies like Yang *et al*. [15]. These analyses may enable actionable solutions for public health officials once consistent identification of contributing predictors is achieved.

Although the GLM appears to show promise with its simultaneous ability to perform ancestral state reconstruction and also assess the contribution of predictor variables of interest, there has yet to be an assessment of how a standard BSSVS approach and a GLM approach differ in reconstructing a phylogeny. Specifically, no study has yet compared root state probabilities in a phylogeny constructed via BSSVS to the same probabilities using the GLM approach. Such information may inform researchers of differences in phylogeographic trends that may be experienced by choosing one framework over the other. In this work we analyze the 2014–15 H3N2 flu season within the U.S. by performing ancestral state reconstruction of a discrete location variable via the following three frameworks: an asymmetric substitution model without BSSVS (–BSSVS), an asymmetric substitution model with BSSVS (+BSSVS) [1], and a GLM [12]. For the BSSVS framework, we analyze separate versions that place both a Poisson distribution (+BSSVS(P)) and a uniform distribution (+BSSVS(U)) on the number of rate parameters that achieve a point-mass on 1.0 in order to determine the influence of location priors. For the GLM framework, we analyze separate versions that include and do not include sample size predictors, which we denote as GLM(+SS) and GLM(–SS), respectively, in order to directly quantify the effect of sampling bias on GLM-constructed rate matrices and potential suppression of the signal of other predictors. This brings us to a total of five methods that encompass the three frameworks. We refer readers to Materials and methods for full details on the methods. These selections allow us to empirically evaluate differences in phylogenies obtained via each method and to determine whether one framework provides more accurate posterior estimates given a fixed set of data. We demonstrate these trends using multiple random samples from a large collection of flu sequences to show reproducibility as well as analyze several coalescent tree priors to show consistency among the reconstruction methods across varying parameters. Finally, we show that support for GLM predictors can change given the tree priors and sequence sets, but that trends among specific predictors will emerge to allow accurate determination of their impact on viral diffusion.

## Results

**Fig 1:**
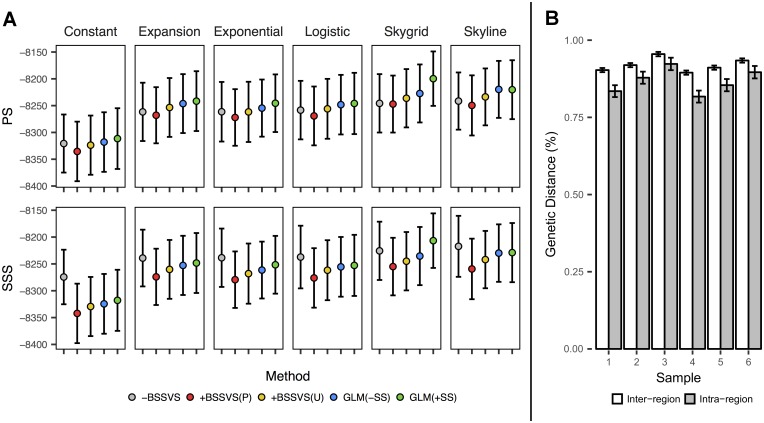
Model comparison statistics and location-specific genetic diversity. (A) Model comparisons obtained via path sampling (PS) and stepping stone sampling (SSS) for the six coalescent priors and five methods. (B) Average genetic distances between all pairwise intra-region and inter-region sequences for the six samples, expressed as a percent, with 95% confidence intervals shown as error bars.

In Fig 1A, we show mean log marginal likelihood estimates among the six samples obtained by path sampling (PS) and stepping stone sampling (SSS) for each prior and reconstruction method. For PS, the two best-performing mean methods are the GLM(+SS) and GLM(-SS), respectively, under each prior. The mean +BSSVS(U) outperforms the mean +BSSVS(P) under each prior as well, although the mean -BSSVS exceeds both under the constant and exponential priors. For SSS, the log marginal likelihood increases in a near-linear manner for the +BSSVS(P), +BSSVS(U), GLM(–SS), and GLM(+SS) methods. The -BSSVS method, however, finds the largest posterior support under the constant, expansion, exponential, logistic, and Skyline priors.

In S1 Fig, we present log marginal likelihood estimates for each individual model. From S1 Fig, we show that each GLM(+SS) and GLM(–SS) unanimously finds more posterior support than their corresponding +BSSVS(P) for both PS and SSS. The +BSSVS(P) method demonstrates consistently poor performance, as its posterior estimates are the worst of the five methods in 25 of 36 PS analyses and 32 of 36 PS analyses (79% overall) across all priors, while no GLM(+SS) or GLM(–SS) yields the lowest posterior estimate of model support among the three methods for either PS or SSS under any prior, although no pairwise t-test shows a significant difference.

Each of the 180 models show statistically significant differences between the null and observed means for the association index (S2 Fig). These data suggest stronger support for the phylogeny-trait association [16] and, as all p < 0.01, suggest the evolution of influenza during this flu season was structured by geography. The support of the sampling location-phylogeny associations observed in S2 Fig can be explained, in part, by the amount of genetic diversity observed within and across each region. In Fig 1B we show the average genetic distances between intra-region and inter-region sequences. Here, we calculated the genetic distances among all $\left(\begin{array}{c} 285\\ 2 \end{array}\right)$ pairwise sequences and present the mean distance of sequences sampled in the same region (e.g. Region 1-Region 1) to those sampled in different regions (e.g. Region 1-Region 2). From Fig 1B, the pairwise intra-region sequences (n = 4,496 per sample) have a lesser amount of genetic diversity than the pairwise inter-region sequences (n = 35,974 per sample) in each our six sequence sets. A two-tailed t-test shows p < 0.01 for each sample, indicating that sequences from within the same region demonstrate significantly lower amounts of genetic diversity than those from external regions. The average intra- and inter-region distances in the full set of 1,163 sequences are 0.872% (95% CI = [0.867, 0.878]), and 0.929% (95% CI = [0.926, 0.932]), respectively (p < 0.0001). These data demonstrate that our method of downsampling maintained representative levels of genetic diversity across the six samples.

**Fig 2:**
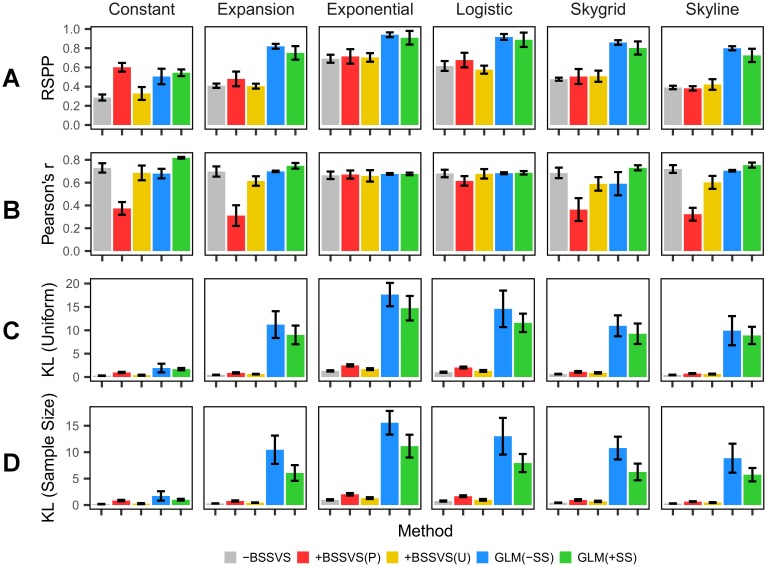
Mean posterior metrics of the MCC phylogenies. Values represent the mean indicated statistic from the six samples under each coalescent prior and method with error bars representing the standard error. (A) Root state posterior probability. (B) Pearson’s correlation coefficient for the number of sequences per discrete state and the root state posterior probability for each discrete state in each model. (C) Kullback-Leibler divergence calculated assuming a uniform prior probability per discrete state. (D) Kullback-Leibler divergence calculated assuming a prior probability proportional to the number of sequences per discrete state.

In Fig 2, we show four root state metrics obtained from the maximum clade credibility (MCC) trees of each of the 180 models. In Fig 2A, we show the mean root state posterior probability (RSPP). Aside from the constant coalescent prior, the mean GLM(–SS) and GLM(+SS) methods consistently show the largest mean RSPP of the five methods. The mean GLM(–SS) finds significantly greater RSPPs under each coalescent prior than the mean -BSSVS (p < 0.03 for each coalescent prior) and significantly greater RSPPs than both the mean +BSSVS(P) and +BSSVS(U) for the expansion and exponential coalescent priors. Similarly, the GLM(+SS) shows a mean RSPP significantly greater than the -BSSVS and +BSSVS(U) methods for all coalescent priors except constant, and significantly greater RSPP than the +BSSVS(P) for the constant, expansion, Skygrid, and Skyline coalescent priors. Across all coalescent priors, the mean RSPP for the -BSSVS, +BSSVS(P), +BSSVS(U), GLM(–SS), and GLM(+SS) methods are 0.48, 0.56, 0.49, 0.81, and 0.74 respectively, These differences per method could be influenced by the sample size per discrete state, so we show the Pearson’s r correlation coefficient between the sample size at each discrete state and its corresponding posterior probability at the root in Fig 2B. Here we observe that the +BSSVS(P) shows a correlation coefficient less than 0.4 for the constant, expansion, Skygrid, and Skyline coalescent priors but for the exponential and logistic coalescent priors the coefficient is nearly doubled. Meanwhile, the +BSSVS(U), -BSSVS, GLM(–SS), and GLM(+SS) methods are generally consistent under all priors. The mean +BSSVS(P) shows significantly less correlation than each of the other four methods for the constant, expansion, and Skyline coalescent priors (p < 0.02 for each) while the +BSSVS(U), -BSSVS, and GLM methods do not show any significant differences under any coalescent prior.

Fig 2C and 2D show the Kullback-Leibler (KL) divergence between the prior and posterior probabilities at the root states calculated using two different prior assumptions (see Materials and methods for details). KL values indicate the extent to which a model is able to generate different posterior probabilities at the root state from the prior probabilities at the root state. That is, high KL values indicate strong divergence from the prior probabilities and, thus, strong posterior information gain, while low KL values indicate the opposite. From Fig 2C and 2D, the mean GLM(–SS) and GLM(+SS) KL divergences demonstrate a marked increase over the -BSSVS, +BSSVS(P), and +BSSVS(U) methods under the expansion, exponential, logistic, Skygrid, and Skyline coalescent priors (p < 0.02 for all two-tailed t-tests. Under the constant coalescent prior, both the mean GLM(–SS) and GLM(+SS) KL divergences exceed the mean KL under both assumptions of the -BSSVS, +BSSVS(P), and +BSSVS(U) methods, but none of these values are significant. The +BSSVS(P) method, in turn, shows significantly greater KL divergences under both assumptions than the -BSSVS method under all coalescent priors and than the +BSSVS(U) method under the constant, exponential, and logistic coalescent priors. We show data for each of the four metrics in Fig 2 by individual model in S3 and S4 Figs.

We summarize the identified root states of the four methods in Table 1. Here, we can see that the -BSSVS method identified three different regions, with the majority occurring in Region 4, while Region 5 is identified in over 30% of -BSSVS models. The +BSSVS(P) method identified six different regions as the root state, with Regions 6 and 4 representing the most frequently-identified. The +BSSVS(U) method identified Region 4 in nearly half of the models while Regions 5 and 6 account for the remainder of models. Comparatively, 35 of the 36 GLM(–SS) runs identified Region 4 as the root state, with the lone exception being Sample 2 using the Skygrid coalescent prior, which identified Region 8. For the GLM(+SS) analyses, Region 4 is identified as the root state in 33 of 36 models while Region 5 accounts for the remaining three. The root heights and corresponding Bayesian credible intervals are similar between the three methods for each sample and each coalescent prior (S5 Fig).

**Table 1:** Frequencies of the root states identified in the MCC tree under each reconstruction method.

Method	State									
	1	2	3	4	5	6	7	8	9	10
–BSSVS	–	–	–	23	11	2	–	–	–	–
+BSSVS(P)	–	2	1	10	6	16	–	1	–	–
+BSSVS(U)	–	–	–	17	10	9	–	–	–	–
GLM(–SS)	–	–	–	35	–	–	–	1	–	–
GLM(+SS)	–	–	–	33	3	–	–	–	–	–

**Fig 3:**
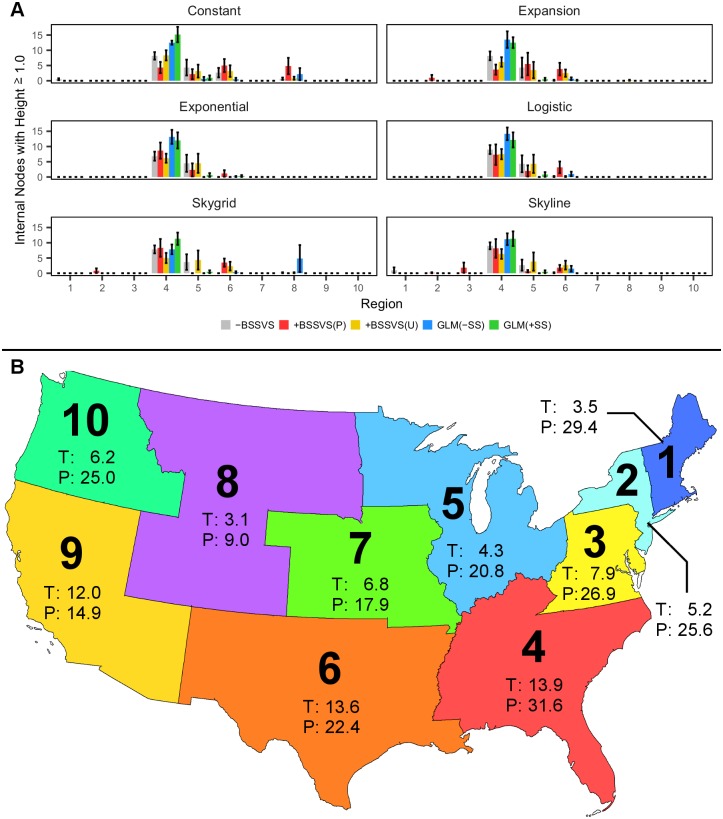
Geographic trends in coalescent events. (A) The number of internal nodes with a height of at least one year in age (NH1s) under each method and for each coalescent prior. Bars represent the average number of such nodes across all six samples, with error bars representing the standard error. (B) Map of the contiguous U.S., colored by the ten discrete states used in this study. Each region is annotated with its average temperature (T, in °C) and precipitation (P, in cm) during the September—May months. Temperature and precipitation data represent the point estimates used in our GLMs for those respective predictors.

As influenza viruses rarely persist for more than one season, except in tropical areas [17, 18], we obtained the geographic distribution of the number of internal nodes with a height of at least one year (NH1s) from the MCC tree of each model and show these data in Fig 3A. From Fig 3A, we can see that the -BSSVS method indicates that Region 4 contains the highest volume of NH1s under each prior, while Region 5 contains the second-largest volume of NH1s. The +BSSVS(P) method shows Region 4 containing the most NH1s for the exponential, logistic, Skyline, and Skygrid coalescent priors, with Region 6 accounting for the next largest volume in the latter three priors. Under the constant coalescent prior, a nearly equal amount of NH1s are observed in Regions 4, 6, and 8, while the expansion prior shows Region 5 containing the largest number of NH1s. For the +BSSVS(U) method, the NH1s are most commonly observed in Region 4 under each coalescent prior, with Regions 5 and 6 primarily accounting for the remaining nodes. The frequency of NH1s in Region 8 are low under this method, but do occur under the constant, expansion, and Skygrid coalescent priors. Finally, the NH1s are largely concentrated in Region 4 for both the GLM(–SS) and GLM(+SS) methods under each coalescent prior.

**Fig 4:**
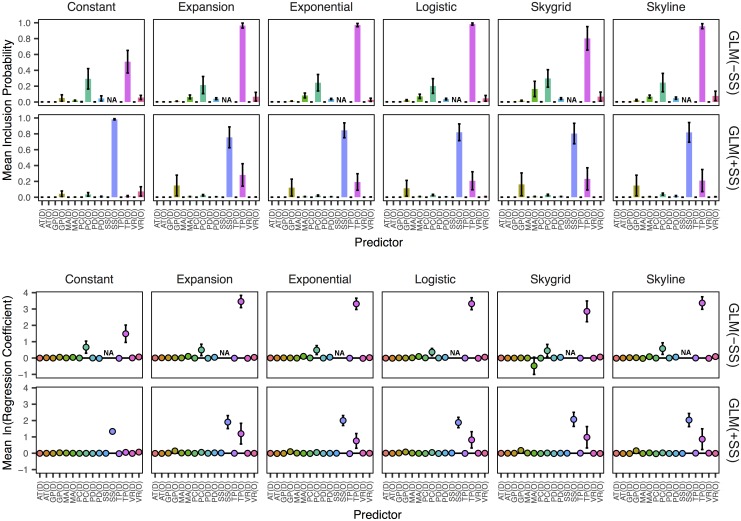
Mean posterior estimates of supported predictors. We show the inclusion probabilities and regression coefficients for all predictors for both the GLM(–SS) and GLM(+SS) analyses. Point estimates represent the mean of each statistic across the six models for each prior, with error bars representing the standard error of these estimates. Predictor abbreviations are: air travel (AT), glycoprotein content (GP), median age (MA), precipitation (PC), population density (PD), sample size (SS), temperature (TP) and vaccination rate (VR).

The frequent identification of Region 4 as the root state (Table 1) and location of NH1 events (Fig 3A) indicates that there is likely at least one local variable playing a role in the tree topologies. Given this, from Fig 3B we note that Region 4 exhibits both the highest expected temperature and precipitation during a typical flu season as we compare the posterior support of all predictors for both the GLM(–SS) and GLM(+SS) methods in Fig 4.

From Fig 4, we can see that sample size at the region of origin (SS(O)) is strongly supported for the GLM(+SS) runs with Bayes factor (BF) > 69 for each coalescent prior and with each corresponding mean regression coefficient greater than 1.33. The predictor with the second largest support for inclusion in the GLM(+SS) runs is temperature at the region of origin (BF > 5 and regression coefficient > 0.75 for each prior except constant size), followed by glycoprotein at the region of origin (3.0 < BF < 4.5 for the expansion, exponential, Skyline, and Skygrid coalescent priors) although the respective mean regression coefficients for glycoprotein remain near zero. For the GLM(–SS) runs, temperature at the region of origin yields the largest mean posterior inclusion probability across all coalescent priors (BF > 20 for each prior, BF > 400 for the expansion, exponential, logistic, and Skyline priors) followed by precipitation at the region of origin (5.0 < BF < 8.5 for all priors). Mean posterior estimates of the corresponding regression coefficients and their standard errors, shown as E(β|δ = 1), indicate strictly positive values for these two predictors in the GLM(–SS) runs, although the 95% highest posterior density (HPD) of the regression coefficient for precipitation at the region of origin spans zero for each model (S6 Fig). If the entire HPD lies on the positive side of zero, this suggests that the predictor is driving the diffusion of the virus. Conversely, if the entire HPD lies on the negative side of zero, this suggests that the predictor is rather preventing the diffusion. Thus, we show the proportion of GLMs in which the absolute value of the HPD is positive in Table 2. The 95% HPDs of temperature at the region of origin are strictly positive in 26 of the 36 GLM(–SS) runs and span zero in the remaining ten. The glycoprotein predictor at the region of origin finds the highest mean support for the constant prior (BF = 1.1), which is a sharp turn from the GLM(+SS) runs. See Materials and methods for more information on metrics of support and interpretations of our predictors. We show the posterior regression coefficients and inclusion probabilities of every predictor from each of the 36 GLM(–SS) runs in S6 and S7 Figs, respectively, and corresponding data for the 36 GLM(+SS) runs in S8 and S9 Figs, respectively.

**Table 2:** Frequency of GLM predictor support.

Method	Criterion	Predictor at the Region of Origin							
		AT	GP	MA	PC	PD	SS	TP	VR
GLM(–SS)	BF ≥ 3	–	3%	25%	36%	3%	NA	94%	19%
GLM(+SS)	BF ≥ 3	–	17%	–	3%	–	97%	36%	3%
GLM(–SS)	|95% HPD (β)| > 0	–	–	–	–	–	NA	72%	–
GLM(+SS)	|95% HPD (β)| > 0	–	3%	–	–	–	61%	8%	–

Values represent the percentage of models that show BF support for a predictor and the percentage of 95% HPD intervals of the regression coefficient that do not span zero. Predictor abbreviations are: air travel (AT), glycoprotein content (GP), median age (MA), precipitation (PC), population density (PD), sample size (SS), temperature (TP) and vaccination rate (VR).

## Discussion

In this paper, we compared three ancestral state reconstruction frameworks and five total methods using six randomly-drawn sequence samples and six coalescent priors for a total of 180 models while fixing the nucleotide substitution process for each. We compared each of our analyses with established model selection techniques [19, 20] and compared features of each model’s MCC tree to identify posterior statistical support and discrepancies in the phylogeographic reconstructions. Regarding model selection, we found that PS shows the most posterior support for either the GLM(–SS) or GLM(+SS) in 34 of 36 runs (with one -BSSVS and one +BSSVS(U) accounting for the remaining two), while SSS shows the most support for 29 of 36 –BSSVS models, five GLM(+SS), one GLM(–SS), and one +BSSVS(U). Each GLM(–SS) and GLM(+SS) outperformed its corresponding +BSSVS(P) under both PS and SSS. Both statistics agree that +BSSVS(P) models offered the poorest posterior support, as 72% of PS analyses and 89% of SSS analyses (81% combined) show the +BSSVS(P) model as the least-supported among the five frameworks (Fig 1A and S1 Fig), although we note that no framework shows significantly more support than any other framework for PS or SSS via t-tests.

Although the -BSSVS method is highly supported under SSS, the method fails to find strong support regarding both RSPP and KL divergence (Fig 2C, 2D and S4 Fig). The RSPPs using the -BSSVS method are significantly lower than those obtained via the GLM(–SS) method (p = 0.03 for the constant coalescent prior, p < 0.001 for the expansion, exponential, logistic, Skygrid, and Skyline coalescent priors), while the GLM(–SS) also show a significant increase for KL divergence for both the uniform and sample size assumptions over the -BSSVS models under each coalescent prior except for constant size. Similarly, the GLM(+SS) method shows significantly greater RSPPs and both KL divergences than the -BSSVS models (p < 0.03 for all coalescent priors except constant). Meanwhile, the +BSSVS(P) method finds significantly greater RSPPs than the -BSSVS method under only the constant coalescent prior (p < 0.001) and significantly greater KL divergences over the -BSSVS method under each coalescent prior, each with p < 0.03. The +BSSVS(P) method also found significantly greater KL divergences for the constant, exponential, and logistic coalescent priors. The +BSSVS(U) method only found significantly greater support over the -BSSVS method via KL with the sample size assumption for the expansion coalescent prior. While these results show that the -BSSVS method finds poor statistical support at the identified root state, we also found that both the GLM(–SS) and GLM(+SS) methods in turn significantly outperformed both the +BSSVS(P) and +BSSVS(U) models both for both KL divergences under five of the six coalescent priors (excluding constant). The GLM(–SS) runs also found significantly greater RSPPs than the +BSSVS(P) and +BSSVS(U) under each coalescent prior except constant, while the GLM(+SS) runs found significantly greater RSPPs than the +BSSVS(P) and +BSSVS(U) methods for the expansion, Skygrid, and Skyline priors.

The association index of each model obtained via BaTS (S2 Fig) demonstrate a strong association between sampling location and the phylogeny for each of the 180 models, which suggests that the diffusion was spatially-structured. Some of the phylogeny-location association can be attributed to the smaller amount of genetic diversity in sequences from the same region (Fig 1B), however the statistical significance of the intra- and inter-region genetic distances could not fully account for the differences in RSPP and KL divergence, regardless of the coalescent prior. Furthermore, Region 4 was the most frequently-identified root state for the -BSSVS, +BSSVS(U), GLM(–SS), and GLM(+SS) methods, the second most frequently identified root state for +BSSVS(P) method (Table 1), and was also the location of the most NH1s (Fig 3A). These NH1s are biologically important for seasonal influenza, as these viruses typically experience bottlenecking at this height as part of a sink-source ecological dynamic [17, 21, 22]. As Region 4 experiences the highest temperature and most precipitation during flu season, at 6.9°C warmer and 10.3 cm wetter, respectively, than the remaining nine regions (Fig 3B) we describe it as the most “tropical” in the U.S. during a typical flu season. This provides a well-supported explanation for the observed trends in Region 4, especially under both GLM methods. As the data for the GLM(–SS) and GLM(+SS) runs indicate strong support for temperature at the region of origin (Fig 4), our results would suggest that Region 4 is the most likely origin of each of the six samples using those two methods.

This conclusion, however, is hindered by the strong sampling bias exhibited by the GLM(–SS), and GLM(+SS) methods. These two methods (as well as the -BSSVS and +BSSVS(U)) demonstrate consistently strong, positive Pearson’s r correlation coefficients between the root state posterior probability and sample size at each discrete state, regardless of coalescent prior (Fig 2B and S3B Fig). Furthermore, the inclusion of the sample size predictors in the GLM(+SS) runs shows that sample size at the region of origin is strongly influencing its posterior estimates, with 35 of 36 runs showing BF > 3 and 22 of 36 showing a positive 95% HPD on the regression coefficient (Table 2, S8 and S9 Figs). The mean posterior inclusion probability for the sample size predictor at the region of origin corresponds to BFs of 1317.9, 70.0, 122.9, 102.7, 92.6, and 101.8 for the constant, expansion, exponential, logistic, Skygrid, and Skyline priors, respectively. Given the similarities in RSPP, Pearson’s r, and KL data between the GLM(–SS) and GLM(+SS) runs (Fig 2, S3 and S4 Figs), we believe that sample size is influencing the GLM(–SS) runs to a similar degree, although its BF support cannot be measured. Thus, although it would appear that both GLM methods presented in this paper are providing biologically justifiable and statistically supported evidence regarding the diffusion of this influenza virus over our selected time period, the strong sampling biases give us pause. Instead, the significant decrease in Pearson’s r for the +BSSVS(P) models from the other four methods under the constant, expansion, and Skyline coalescent priors provide more confidence in those data, despite its poor performance with respect to log marginal likelihoods via PS and SSS (Fig 1A and S1 Fig).

We compared the -BSSVS, +BSSVS(P), +BSSVS(U), GLM(–SS), and GLM(+SS) methods for modeling a single discrete trait, sampling location, which highlighted differences in diffusion of seasonal influenza in the U.S. Our results collectively indicate that the GLMs provide the strongest posterior support for MCC metrics of the three ancestral state reconstruction frameworks used in this study, however the strong sampling bias exhibited by that method marginalizes confidence in their reconstructions. As mentioned, the strong support for sample size is consistent with previous studies that used the phylogeographic GLMs [12, 13]. Air travel was previously shown to be a driver of the global diffusion of H3N2 using a GLM [12], but none of the GLM(–SS) or GLM(+SS) runs showed support for this predictor. However, our study was performed within a single country and aggregated all air travel data from each individual state into a matrix of region-to-region passenger flux, which perhaps limits its contribution to these models. Furthermore, the paper by Lemey *et al*. [12] discretized by “air communities” (p. 2) to better reflect trends in air travel, while we partitioned strictly based on pre-defined, arbitrary geographic regions. We also assumed a single introduction into the U.S. and did not include incoming travel from international flights that could certainly have introduced strains with more genetic diversity than those used in this study.

We recognize several limitations with this study including the omission of international air travel. In addition, our assumption of a single introduction into the U.S. could also have limited inference regarding the contribution of air travel and may explain the lack of BF support for that predictor from both region of origin and destination when a previous study has implicated these data as a driver of the diffusion [12]. Also, the transportation predictor fails to incorporate inter-region travel via ground transportation, which certainly could have implications within a single country. Furthermore, we only analyzed hemagglutinin sequences in this study and did not investigate neuraminidase or any other segments of the influenza genome. We arbitrarily selected 25% of samples from each region for our subsampling in order to better reflect the observed sampling frequencies, but it is possible that larger subsample sizes or an alternative sampling approach could have resulted in stronger or weaker support for the predictors in the GLM as well as the RSPPs via the three reconstruction approaches. However, our use of Pearson’s correlation coefficient between sample size and root state posterior probability (Figs 2B and 3B) and comparison of GLMs that include and do not include sample size predictors aim to outline the impact of sampling bias within our dataset. We plan to conduct similar research on additional influenza seasons and using alternative sampling methods in order to further study whether this sampling bias is a systematic function in the GLMs or is limited to the dataset used in this study. Sampling bias is a known issue in phylodynamics [23, 24] and may not be possible to eliminate, although varying approaches may differ in their sensitivity to such biases. Finally, we limited our study to a single influenza season which prevents seasonality comparisons and impacts from local persistence.

Overall, this study aimed to investigate the phylogeography of the H3N2 influenza viruses that circulated in the U.S. during the 2014–15 flu season and to also investigate three established methods of ancestral state reconstruction. While our GLM results provide superior posterior support than either +BSSVS method or the -BSSVS framework, these results appear to be dominated by a strong sampling bias. Although these results are not necessarily incorrect, the investigation of additional frameworks reveals that the +BSSVS(P) is likely the “best” approach for this dataset to minimize such concerns, depending on the selection of coalescent prior, if given the choice among the five presented in our work for this particular virus and time frame. Furthermore, we demonstrate that our approach of subsampling to compare multiple models may not only reflect subtle changes to the phylogeny but also to the contribution of the predictor variables in the GLMs. Although we do not believe that the GLM provides an ideal, unbiased reconstruction framework for our dataset, this type of assessment could be valuable for understanding the true nature of the phylogeny-sampling location association in future work. Such studies may also encourage researchers to utilize the GLM framework as a means of obtaining more information-driven variables into their phylogeographic studies and to unlock the potential for more accurate ancestral state reconstructions to better aid epidemiological and public health efforts.

## Materials and methods

### Sequence and model setup

#### Nucleotide sequences

We used the EpiFlu database from the Global Initiative for Sharing All Influenza Data (GISAID) to collect H3N2 hemagglutinin (HA) sequences from the 2014–15 flu season. We obtained our dataset on 2015-10-16 using the following search terms: Host = *Human*, Location = *United States*, Collection Date = *2014-09-29 to 2015-05-17*, Submitting Laboratory = *[United States*, *Atlanta] Centers for Disease Control and Prevention*, Required Segments = *HA*, Min Length = *1*,*659*. This search resulted in 1,220 sequences, and we further eliminated sequences from Alaska, Hawaii, and the District of Columbia and those that did not have a specific state listed to obtain a final set of 1,163 sequences. In order to reduce the size of the transition rate matrix, we discretized the states into the ten U.S. Department of Health and Human Services (HHS) regions [25], which we show in Fig 3B.

#### Ancestral state reconstruction methods

Our phylogeographic assessment assumes that geographic sampling traits follow a continuous-time Markov chain (CTMC) process along the branches of an unknown phylogeny that is informed through sequence data. The models we compare differ in how one parameterizes the infinitesimal rates of the among-location CTMC process. Here, we first parametrized the discrete location trait with a basic asymmetric substitution model (–BSSVS). Next, following Lemey *et al*. [1], we retained the asymmetric substitution model but specified a truncated Poisson prior on the number of non-zero rates (+BSSVS(P)). Here, 50% of the prior probability lies on the minimal rate configuration (i.e. nine non-zero rates connecting the ten HHS regions). Similarly, we also placed a uniform probability on the location prior in order to test the effects of the selected location prior on the BSSVS procedure +BSSVS(U). We compare the -BSSVS and +BSSVS(P) methods with recent developments in virus phylogeography that have advanced modeling of among-location transition rates as a log-linear GLM of predictors of interest [12]. Here, we followed this framework and parameterized GLMs with seven demographic, environmental, and genetic factors that we take from both region of origin and region of destination for a total of 14 predictors in the GLM(–SS) runs. In the GLM(+SS) runs we also include an additional two sample size predictors for a total of 16 predictors. This approach yields a quantifiable assessment of the inclusion and contribution of each predictor variable to the overall transition rate matrix between our ten locations by estimating posterior probabilities of all 2^14^ or 2^16^ possible linear models via a BSSVS procedure. We specified a 50% prior probability that no predictor will be included to enable calculation of Bayes factors (BFs) as a metric of support for the inclusion or exclusion of any given predictor. Here, we consider any predictor with BF > 3.0 to be supported for inclusion. For further details on the underlying theory and mathematical definitions of this GLM approach, we refer readers to Lemey *et al*. [12].

#### Summary of rate parameters

For both the -BSSVS and +BSSVS frameworks, there are K(K–1) relative rate parameters where K = 10 discrete states for our dataset [1]. For the -BSSVS framework, these rate parameters are each a priori independently gamma distributed with scale and shape parameters of 1.0, and for the +BSSVS framework these rate parameters are each a priori with a mixture of point-mass on 1.0 and on the same gamma distribution as the -BSSVS rate parameters. The number of parameters that achieve the point mass on 1.0 for the +BSSVS framework are Poisson distributed with a mean of 9.0 (for the +BSSVS(P) method) and uniformly distributed for the +BSSVS(U) method. For the GLM framework, there are 14 and 16 regression parameters (i.e. predictors) for the GLM(–SS) and GLM(+SS) methods, respectively, as outlined below. The regression parameters are each a priori in part a mixture of point-mass on 0 and in part normally distributed with a mean of 0 and a variance of 4.0 [12].

#### Sequence subsampling

In order to investigate the effects of sampling biases, we performed multiple analyses using random samples from our full set of 1,163 sequences. We created six independent sequence samples by selecting 25% of the sequences in each region at random without replacement and assume that each is representative of the entire flu season. These samples allow us to reveal whether the three frameworks will agree on the root location, root state posterior probability, height, and other trends in the phylogenies as well as show the reproducibility of the support for our GLM predictor variables. We did not identify any duplicate sequences from the same discrete state in any of the six samples. We aligned these six samples, each of which contained 285 sequences, using MAFFT v7.017 in Geneious Pro v.6.1.8 (Biomatters Ltd., Auckland, New Zealand). We treated each alignment as an independent dataset for our phylogeographic reconstructions and report all GISAID accession numbers and discrete state assignments in S1 Table. The six samples and six coalescent priors result in 180 total models, 36 from each of the -BSSVS, +BSSVS(P), +BSSVS(U), GLM(–SS), and GLM(+SS) methods.

### GLM predictors

#### Human population and age

We obtained population estimates and land area per state from the U.S. Census Bureau (USCB) MAF/TIGER database (https://www.census.gov/). Population data are released annually and represent the population as of 2014-07-01 for the 2014–15 flu season, and we used these values to create a density per region. We also obtained the median age per state from the USCB and used these values as a separate predictor, aggregated by region.

#### Temperature and precipitation

For our climate predictors, we obtained data from the National Climatic Data Center of the National Oceanic and Atmospheric Administration (NOAA). We collected temperature and precipitation data for the 30-year climate normal from 1981–2010 for the 9,359 stations in the contiguous 48 states, not including the District of Columbia. As we are interested in the typical temperatures and precipitations observed during a flu season, we computed the average of all September-October-November, December-January-February, and March-April-May summary datasets from stations in each region. We take these values for temperature (in degrees Celsius) and precipitation (in centimeters) to represent the typical flu season climate for each region.

#### Influenza vaccination rates

We obtained state-level data on the vaccination rates for the 2014–15 flu season from FluVaxView by the Centers for Disease Control and Prevention (CDC) [26] and aggregated them to a region-wide average. These data represent all individuals at least six months of age that received the annual flu vaccine at any point in time during the season.

#### Air travel

In order to account for travel between the ten regions, we obtained data from the Official Airline Guide, Ltd. as the number of seats on domestic flights from each airport each other airport within the contiguous U.S. for the 2012 calendar year. We assumed that the number of seats is proportional to the number of passengers on each flight and that the 2012 travel data is proportional to that of 2014–15. We discretized the data from each individual airport into a total number per HHS region to create a matrix of travel flux. These data do not include flights originating from international locations and thus strictly represent passenger flux among the ten HHS regions used in this study. We held this predictor constant through each of the six samples.

#### Glycoprotein content

Influenza vaccines are designed to induce neutralizing antibodies of both the hemagglutinin and neuraminidase viral surface glycoproteins [27] in order to protect against future infections with similar antigenic properties to the vaccinated strain [28]. The glycoprotein (GP) content of a sampled virus thus provides an indication of the sample’s similarity to the strain vaccinated against during that season. Of the 1,163 sequences in our dataset, 533 (46%) contained metadata regarding the GP content of the sample. The authors annotated these sequences with the binary “LOW GP” or “GP” to represent the similarity of the GP to the A/Texas/50/2012 (H3N2)-like virus strain vaccinated against during the 2014–15 flu season [29]. For each sample, we calculated the proportion of sequences with “LOW GP” to the total sequences with known antigenic content per region as a measure of the circulating strain’s disparity from the strain vaccinated against. This is the only predictor in which the values are not fixed among the six samples.

#### Sample size

Previous phylogeographic studies using GLMs have included and found strong posterior support for sample size at the location of origin and/or the location of destination [12, 13] so we included both as predictors (Table 3) in the GLM(+SS) runs. The GLM(+SS) runs thus contain 16 predictors while the GLM(–SS) run contain 14 predictors.

**Table 3:** Summary statistics of the predictors used in this study for the ten discrete states.

Predictor	Mean	SD	Median	IQR
Population Density (people/mi^2^)	165.9	141.0	143.9	161.3
Median Age (years)	38.0	1.6	37.8	2.0
Vaccination Rate (%)	42.6	3.5	43.2	4.5
Temperature (°C)	7.7	4.1	6.5	6.5
Precipitation (cm)	22.4	7.0	23.7	8.2
Low Glycoprotein Content (%, overall)	88.3	3.7	87.8	3.1
Sample Size *	28.5	11.5	27.5	16
Air Travel **	6.1E+06	6.0E+06	4.1E+06	6.7E+06

* Accession numbers for the samples and location data are provided in S1 Table.

**Air travel represents the indicated statistic among all 90 pairwise region-to-region combinations.

### Influenza phylogeography

#### Molecular clock fitting

We performed a preliminary analysis with Path-O-Gen v1.4 (http://tree.bio.ed.ac.uk/software/pathogen/) which showed that relaxed molecular clocks may have overparameterized our models. We therefore selected a strict molecular clock with a rate of 0.001 substitutions per site per year.

#### Coalescent priors and substitution model

In addition to the three reconstruction methods and six sequence samples, we also investigated six coalescent priors in this study: constant size [30], exponential growth [31], logistic growth [31], expansion growth [31], Bayesian Skyline [32], and Bayesian Skygrid [33]. Thus, we completed 180 individual ancestral state phylogeographic reconstructions, one for each sample/coalescent prior/reconstruction method combination (e.g. Sample 1/constant size/GLM, Sample 1/constant size/+BSSVS(P), Sample 1/constant size/–BSSVS, etc.). We specified an HKY+G [34] substitution model following recent phylogenetic studies of H3N2 [12, 35] and preliminary performance analyses using other substitution models. We enabled each of the six samples to parameterize the diffusion process between HHS regions using the -BSSVS, +BSSVS(P), +BSSVS(U), GLM(–SS), and GLM(+SS) methods. We evaluated each model using the BEAST v1.8.4 software package [36] with a chain length of 100 M, logging estimates every 10,000 steps while specifying a single seed across all models. These methods aim to minimize all sources of variance but the randomly selected sequences, tree priors, and glycoprotein content.

#### Analysis of support for models

We used path sampling (PS) and stepping-stone sampling (SSS) to estimate marginal likelihoods of each model, as this procedure has been shown to be an improvement over harmonic mean estimators [19, 20]. Here, we specify a chain length of 1M with 100 path steps, logging every 1,000 steps. For the GLM predictors, we obtained the mean posterior probability of inclusion, BF support values, and the contribution of each predictor to the log-linear rate matrix. In order to determine the impact of geography on the phylogeny, we utilized Bayesian Tip-association Significance Testing (BaTS) [16]. This application tests the null hypothesis that other than by chance, adjoining tips are not more likely to share the same discrete traits. Here, we used our ten HHS regions as discrete traits to be tested under this null hypothesis.

#### Comparison of phylogenies

We used TreeAnnotator v1.8.4 to construct a maximum clade credibility (MCC) tree for each of the 180 runs after discarding the first 10% of trees as burnin. We viewed and annotated the MCC trees using FigTree v1.4.2 for direct comparison of the ancestral state reconstructions. From each MCC tree, we recorded the root state, root height and its 95% Bayesian credible interval, root state posterior probability, and the location of all nodes with a height exceeding one year. We also calculated the Kullback-Leibler (KL) divergence at the root state of each model. Here, we assumed two different prior probabilities at each discrete state: a uniform prior probability per discrete state (i.e. 0.1 for each of the ten discrete states), and second, a prior probability that is proportional to the number of taxa from that state (e.g. as 26 of 285 taxa were sampled in Region 1 we set its prior probability to 26/285 = 0.0912). The latter approach allows us to account for potential sampling bias in the KL calculations. For several GLMs, we found that the posterior probability of at least one root state was zero, which yields a KL divergence of infinity. In order to present a finite KL value, we assigned these states a posterior probability of 1.0 x 10^−16^ and subtracted this artificial probability from the most probable root state. As an additional step to investigate possible sampling bias, we calculated the Pearson correlation coefficient (r) between the sample size for each of the ten discrete states and its corresponding root state posterior probability for each individual model.

#### Data availability

We have made the XML file and MCC phylogeny for each of our 180 models available for download at https://figshare.com/projects/Magee-Flu-PLoS/16638. We have also made available the six sequence alignments as well as the full set of 1,163 unaligned sequences from which we created our samples.

## Supporting information

S1 Table GISAID accession numbers and discrete states for the 285 sequences in each sample. (CSV)

S1 Fig Model comparisons for the 180 analyses. (A) Log marginal likelihood obtained via path sampling (PS). (B) Log marginal likelihood obtained via stepping-stone sampling (SSS). Metrics are shown for each sample, prior, and method.
(TIFF)

S2 Fig Association index scores obtained via BaTS. For each model, we show the null mean (larger value) and observed mean (smaller value) and their respective 95% confidence intervals. For each model, we observe p < 0.0001 between the null and observed means.
(TIFF)

S3 Fig Individual root state posterior probabilities and potential sampling bias analyses. (A) Root state posterior probability from the MCC tree of each model. The corresponding root state is shown below each bar. See Fig 3B for the locations of these root states. (B) Pearson’s r correlation coefficient between the number of sequences per discrete state and the RSPP for each discrete state in each model.
(TIFF)

S4 Fig Individual Kullback-Leibler divergence statistics of the root state prior and posterior probabilities for each model. (A) Values are calculated assuming a uniform prior probability per discrete state. (B) Values are calculated assuming a prior probability proportional to the number of sequences per discrete state.
(TIFF)

S5 Fig Root heights for the MCC phylogenies. Mean heights are represented by the colored circles with 95% Bayesian credible intervals shown as error bars.
(TIFF)

S6 Fig Posterior regression coefficients of all predictors per sample and prior for the GLM(–SS) runs. Predictor abbreviations are: air travel (AT), glycoprotein content (GP), median age (MA), precipitation (PC), population density (PD), sample size (SS), temperature (TP) and vaccination rate (VR), each evaluated from both region of origin (O) and region of destination (D).
(TIFF)

S7 Fig Posterior inclusion probabilities of all predictors per sample and prior for the GLM(–SS) runs. We consider predictors with inclusion probabilities exceeding the dotted horizontal line, which corresponds to BF = 3.0, to be supported in that model. Predictor abbreviations are: air travel (AT), glycoprotein content (GP), median age (MA), precipitation (PC), population density (PD), sample size (SS), temperature (TP) and vaccination rate (VR), each evaluated from both region of origin (O) and region of destination (D).
(TIFF)

S8 Fig Posterior regression coefficients of all predictors per sample and prior for the GLM(+SS) runs. Predictor abbreviations are: air travel (AT), glycoprotein content (GP), median age (MA), precipitation (PC), population density (PD), sample size (SS), temperature (TP) and vaccination rate (VR), each evaluated from both region of origin (O) and region of destination (D).
(TIFF)

S9 Fig Posterior inclusion probabilities of all predictors per sample and prior for the GLM(+SS) runs. We consider predictors with inclusion probabilities exceeding the dotted horizontal line, which corresponds to BF = 3.0, to be supported in that model. Predictor abbreviations are: air travel (AT), glycoprotein content (GP), median age (MA), precipitation (PC), population density (PD), sample size (SS), temperature (TP) and vaccination rate (VR), each evaluated from both region of origin (O) and region of destination (D).
(TIFF)
