# Supplementary figures and images for: Bayesian phylogeography of influenza A/H3N2 for the 2014-15 season in the United States using three frameworks of ancestral state reconstruction
Source: PLoS Comput Biol. 2017 Feb 7;13(2):e1005389. doi: 10.1371/journal.pcbi.1005389 (PMC5321473; doi:10.1371/journal.pcbi.1005389)

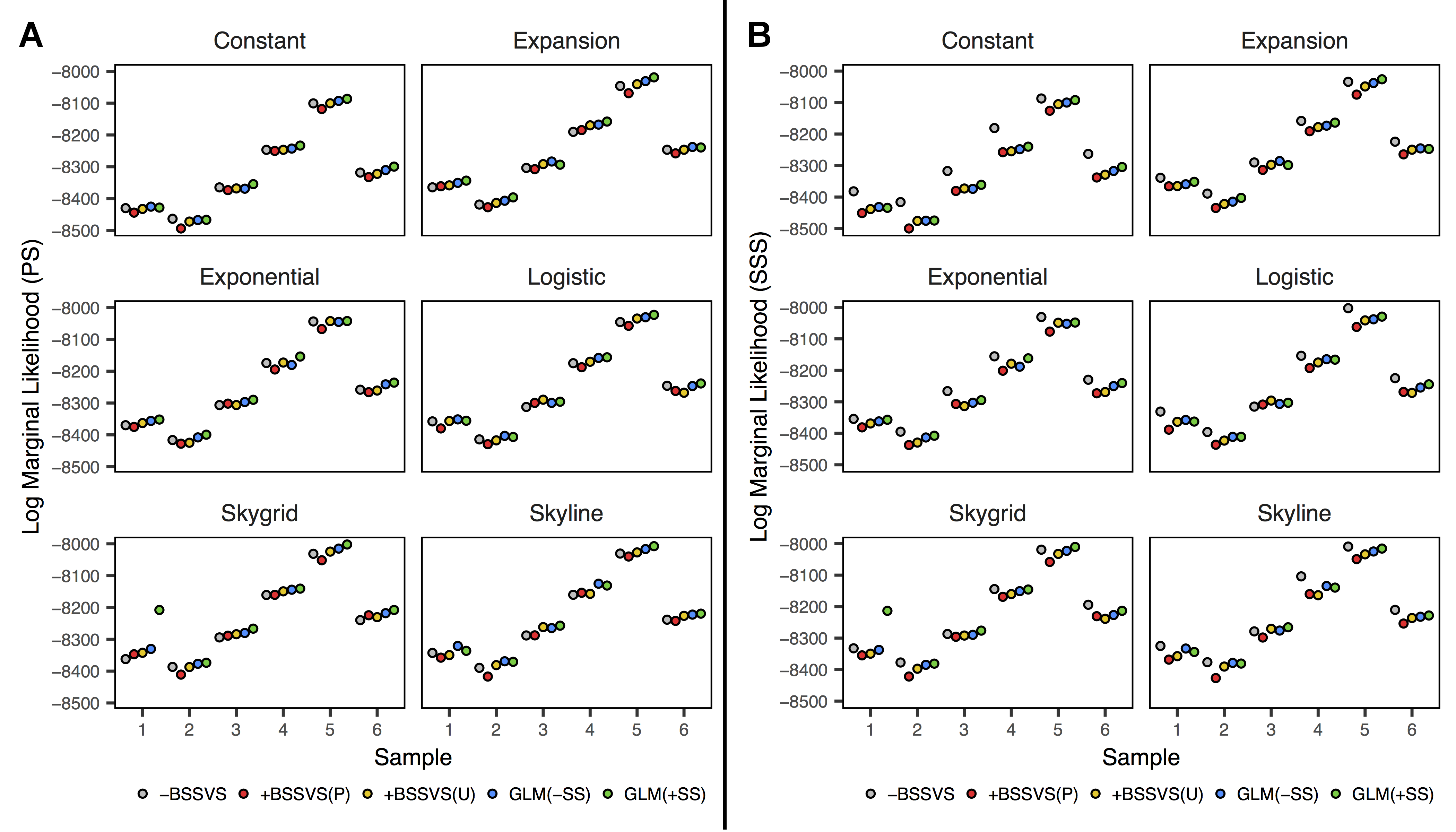

Supplement: S1 Fig — (A) Log marginal likelihood obtained via path sampling (PS). (B) Log marginal likelihood obtained via stepping-stone sampling (SSS). Metrics are shown for each sample, prior, and method. (TIFF) [file pcbi.1005389.s002.tiff]

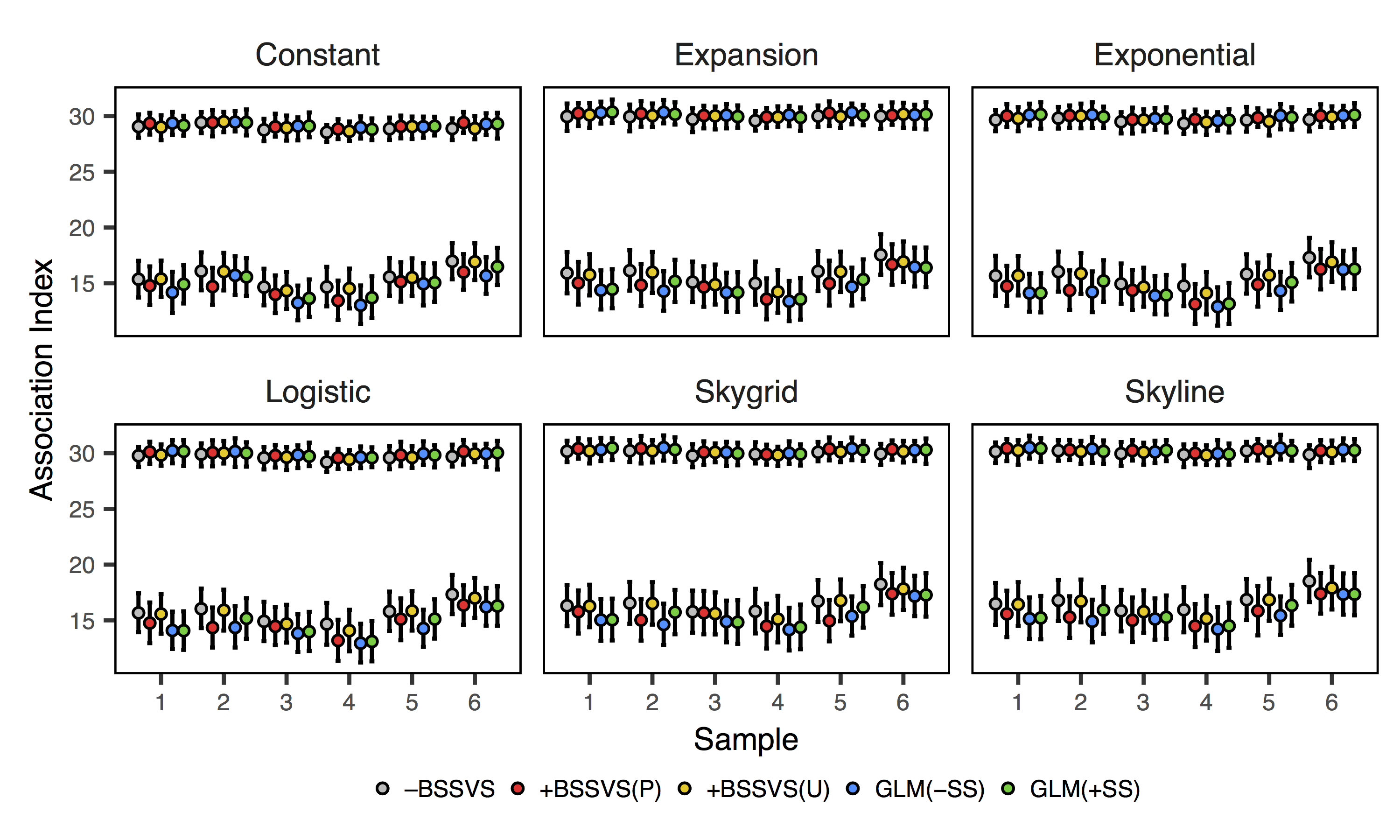

Supplement: S2 Fig — For each model, we show the null mean (larger value) and observed mean (smaller value) and their respective 95% confidence intervals. For each model, we observe p < 0.0001 between the null and observed means. (TIFF) [file pcbi.1005389.s003.tiff]

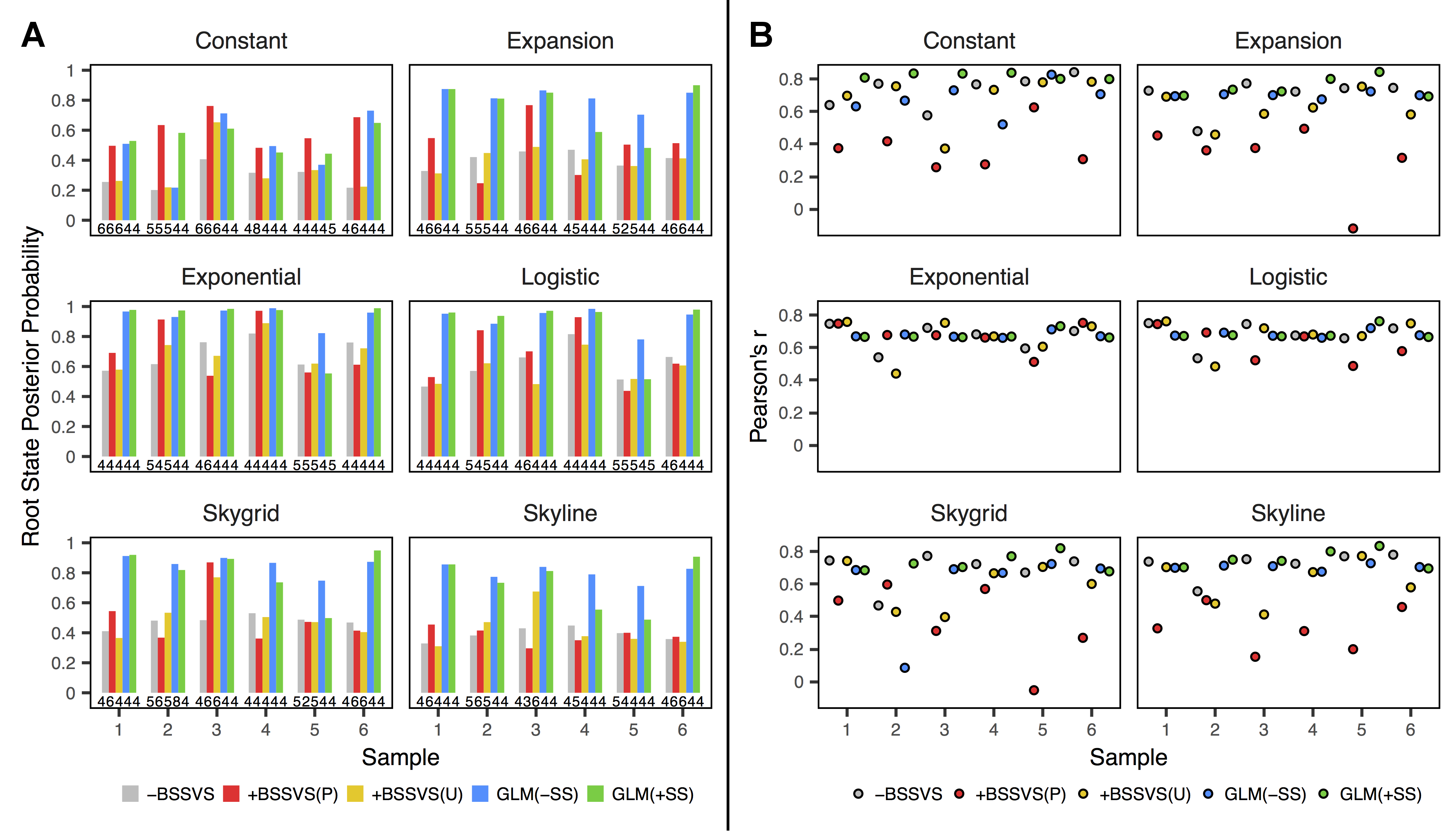

Supplement: S3 Fig — (A) Root state posterior probability from the MCC tree of each model. The corresponding root state is shown below each bar. See Fig 3B for the locations of these root states. (B) Pearson’s r correlation coefficient between the number of sequences per discrete state and the RSPP for each discrete state in each model. (TIFF) [file pcbi.1005389.s004.tiff]

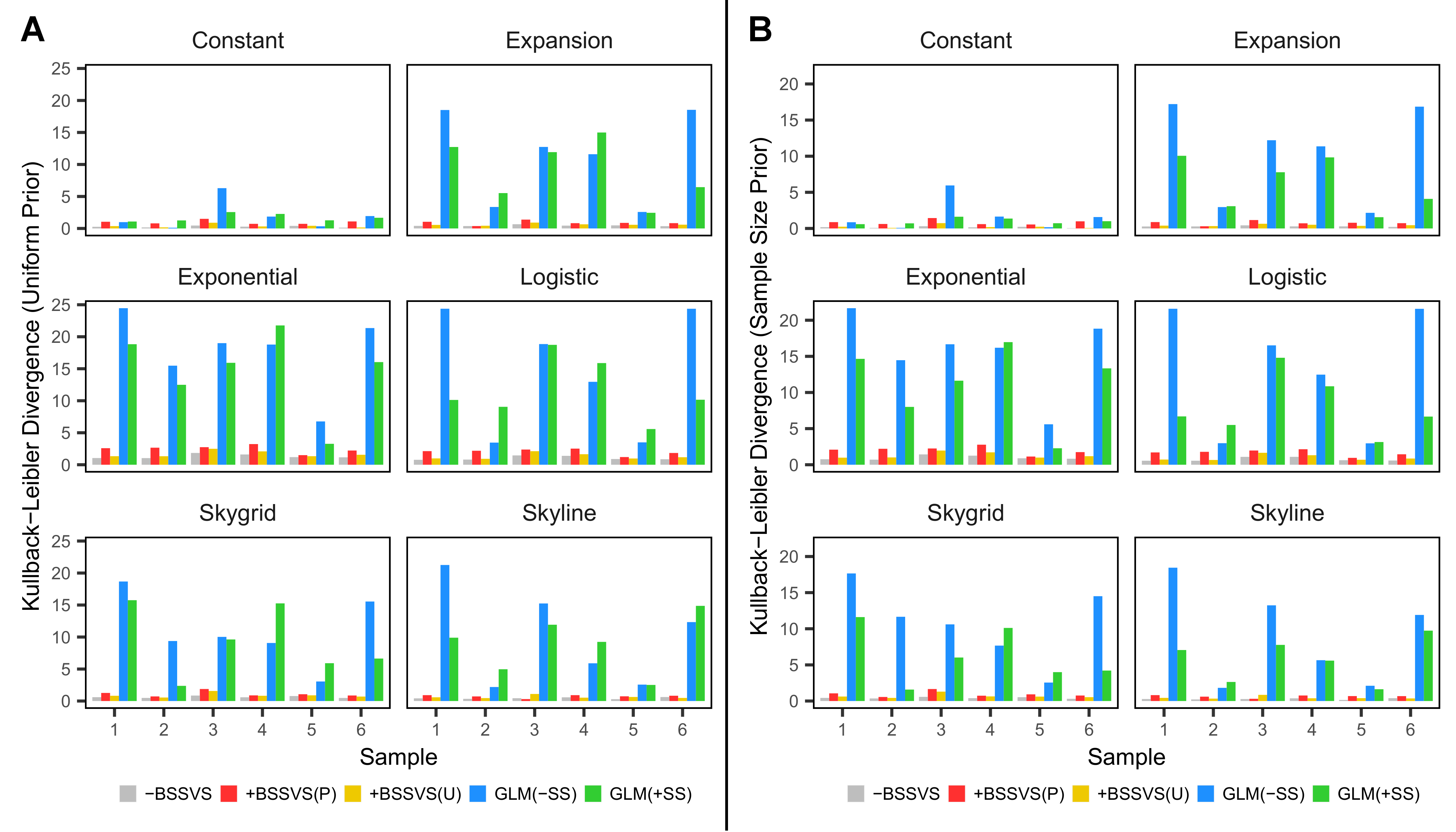

Supplement: S4 Fig — (A) Values are calculated assuming a uniform prior probability per discrete state. (B) Values are calculated assuming a prior probability proportional to the number of sequences per discrete state. (TIFF) [file pcbi.1005389.s005.tiff]

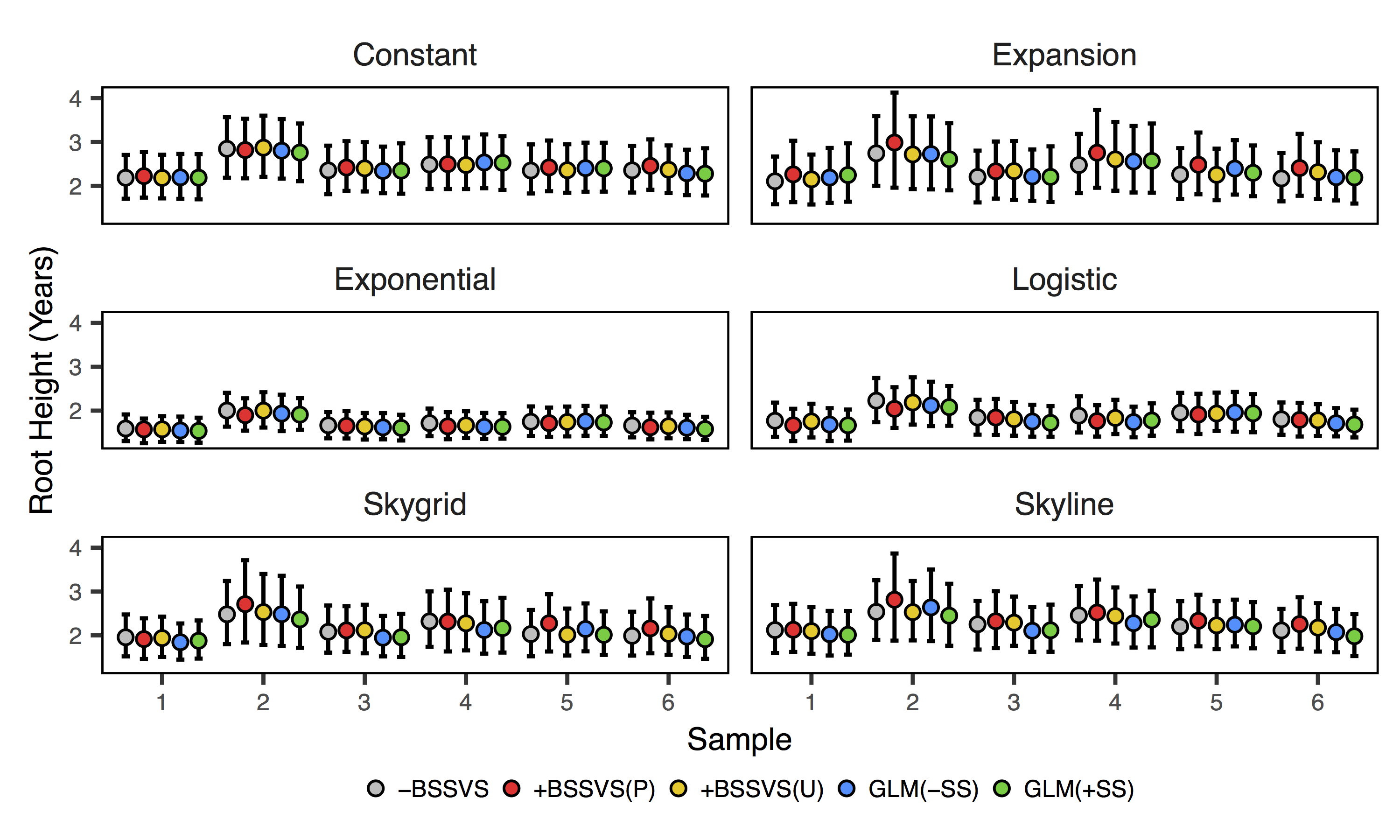

Supplement: S5 Fig — Mean heights are represented by the colored circles with 95% Bayesian credible intervals shown as error bars. (TIFF) [file pcbi.1005389.s006.tiff]

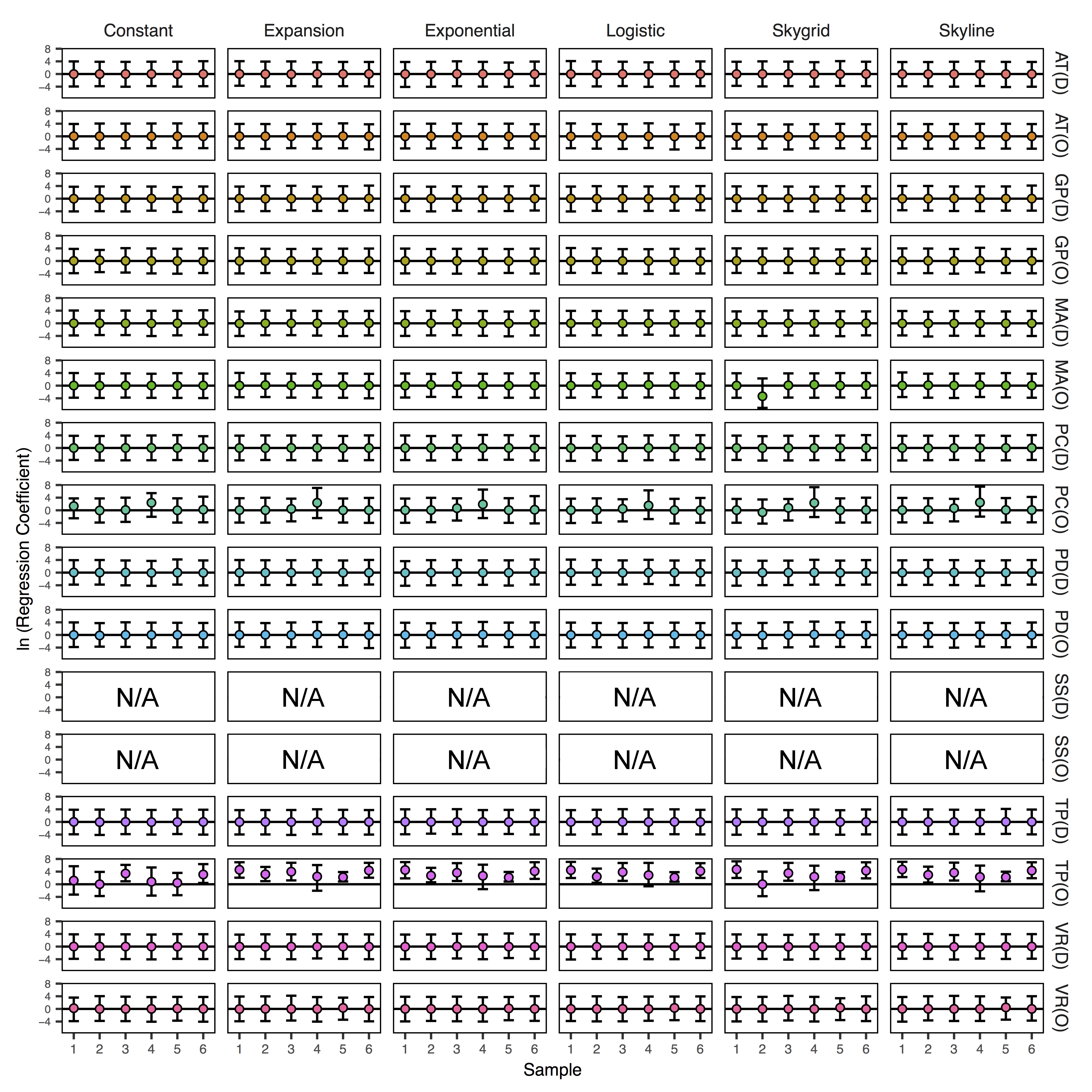

Supplement: S6 Fig — Predictor abbreviations are: air travel (AT), glycoprotein content (GP), median age (MA), precipitation (PC), population density (PD), sample size (SS), temperature (TP) and vaccination rate (VR), each evaluated from both region of origin (O) and region of destination (D). (TIFF) [file pcbi.1005389.s007.tiff]

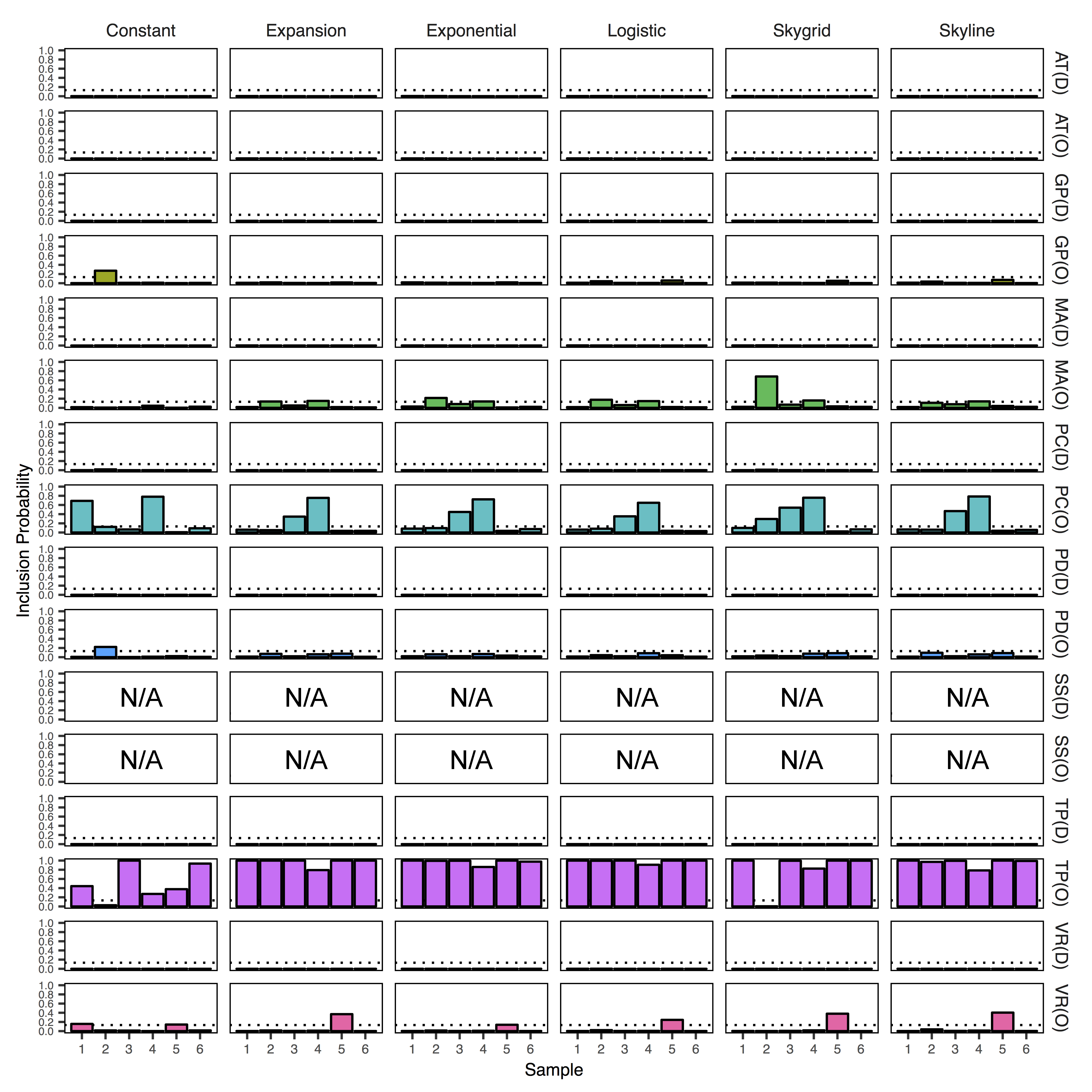

Supplement: S7 Fig — We consider predictors with inclusion probabilities exceeding the dotted horizontal line, which corresponds to BF = 3.0, to be supported in that model. Predictor abbreviations are: air travel (AT), glycoprotein content (GP), median age (MA), precipitation (PC), population density (PD), sample size (SS), temperature (TP) and vaccination rate (VR), each evaluated from both region of origin (O) and region of destination (D). (TIFF) [file pcbi.1005389.s008.tiff]

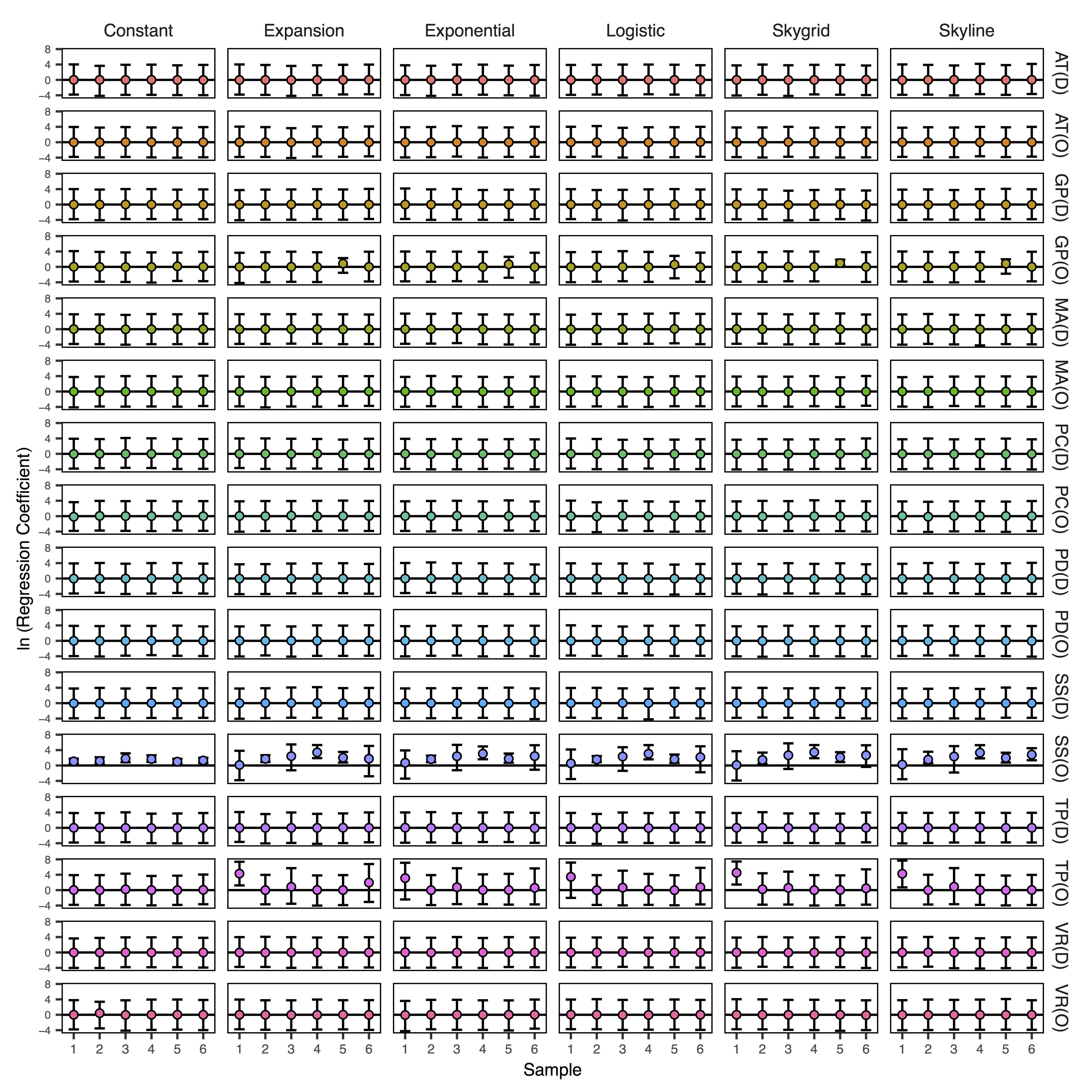

Supplement: S8 Fig — Predictor abbreviations are: air travel (AT), glycoprotein content (GP), median age (MA), precipitation (PC), population density (PD), sample size (SS), temperature (TP) and vaccination rate (VR), each evaluated from both region of origin (O) and region of destination (D). (TIFF) [file pcbi.1005389.s009.tiff]

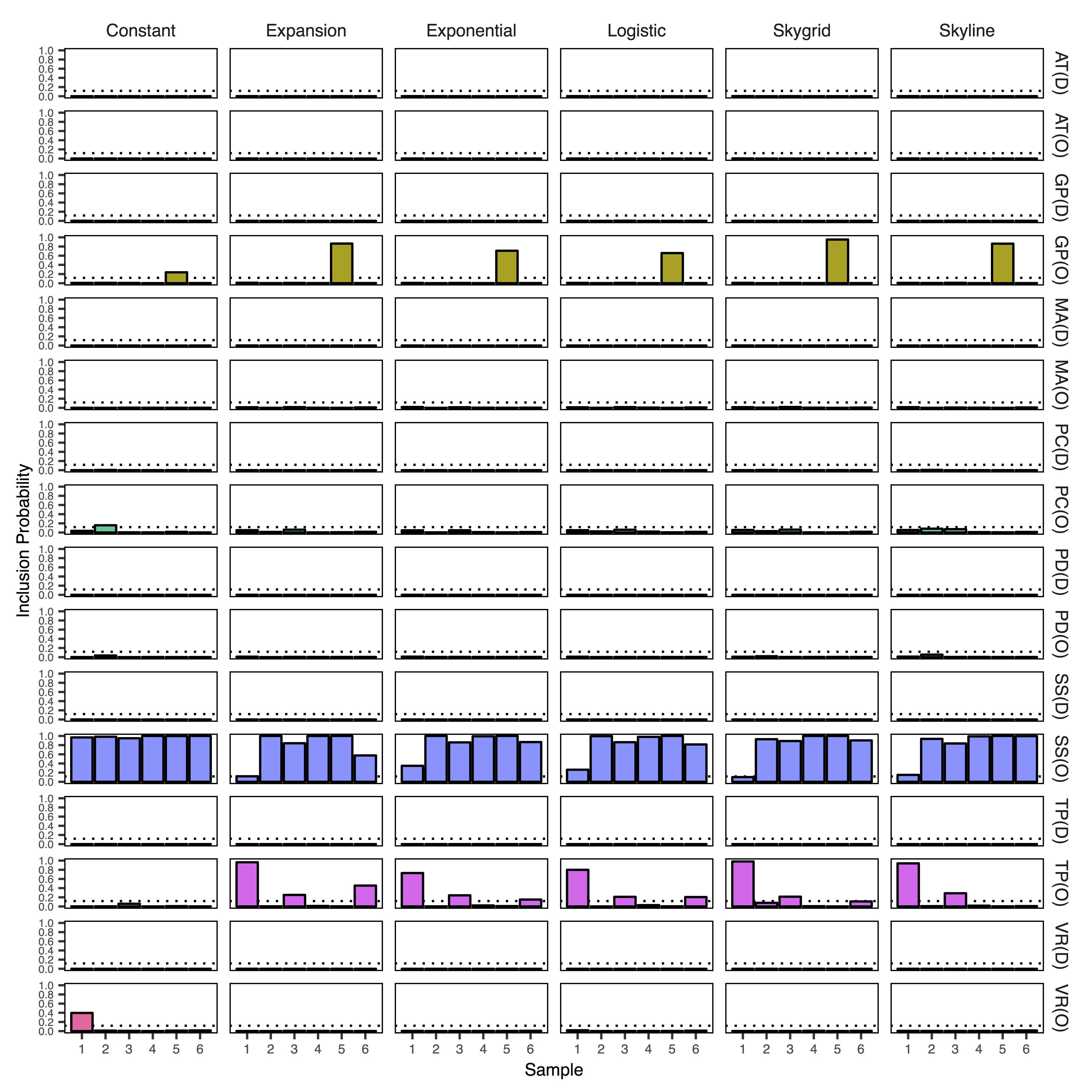

Supplement: S9 Fig — We consider predictors with inclusion probabilities exceeding the dotted horizontal line, which corresponds to BF = 3.0, to be supported in that model. Predictor abbreviations are: air travel (AT), glycoprotein content (GP), median age (MA), precipitation (PC), population density (PD), sample size (SS), temperature (TP) and vaccination rate (VR), each evaluated from both region of origin (O) and region of destination (D). (TIFF) [file pcbi.1005389.s010.tiff]
